# Management of Long-Segment and Panurethral Stricture Disease

**DOI:** 10.1155/2015/853914

**Published:** 2015-12-08

**Authors:** Francisco E. Martins, Sanjay B. Kulkarni, Pankaj Joshi, Jonathan Warner, Natalia Martins

**Affiliations:** ^1^Department of Urology, Hospital Santa Maria, University of Lisbon, School of Medicine, 1600-161 Lisbon, Portugal; ^2^ULSNA-Hospital de Portalegre, 7300-074 Portalegre, Portugal; ^3^Kulkarni Reconstructive Urology Center, Pune 411038, India; ^4^City of Hope Medical Center, Duarte, CA 91010, USA

## Abstract

Long-segment urethral stricture or panurethral stricture disease, involving the different anatomic segments of anterior urethra, is a relatively less common lesion of the anterior urethra compared to bulbar stricture. However, it is a particularly difficult surgical challenge for the reconstructive urologist. The etiology varies according to age and geographic location, lichen sclerosus being the most prevalent in some regions of the globe. Other common and significant causes are previous endoscopic urethral manipulations (urethral catheterization, cystourethroscopy, and transurethral resection), previous urethral surgery, trauma, inflammation, and idiopathic. The iatrogenic causes are the most predominant in the Western or industrialized countries, and lichen sclerosus is the most common in India. Several surgical procedures and their modifications, including those performed in one or more stages and with the use of adjunct tissue transfer maneuvers, have been developed and used worldwide, with varying long-term success. A one-stage, minimally invasive technique approached through a single perineal incision has gained widespread popularity for its effectiveness and reproducibility. Nonetheless, for a successful result, the reconstructive urologist should be experienced and familiar with the different treatment modalities currently available and select the best procedure for the individual patient.

## 1. Introduction

Management of long-segment urethral stricture remains a challenge in reconstructive urology. The surgical treatment of urethral strictures varies according to etiology, location, length, and density of the lesion and fibrosis involving surrounding tissues [1–3]. Treatment of strictures involving the bulbar urethra is relatively well defined and, in most cases, is amenable to excision and end-to-end anastomosis or a short patch onlay substitution urethroplasty [4]. However, long-segment urethral stricture or panurethral stricture disease is less common and the literature on the subject is not abundant.

In the treatment of this condition, several issues must be factored in, such as cause of the stricture, previous urethral surgeries, the quality of the urethral plate, availability of different autologous tissues to be used as flaps or grafts, experience, expertise, and preference of the treating urologist, including his familiarity with tissue transfer techniques [5]. Lichen sclerosus (LS), also known as balanitis xerotica obliterans (BXO), raises specific problems related to treatment, prognosis, and prolonged follow-up [6–10]. The complexity of this condition may require a different dynamic treatment paradigm. However, although a multistage reconstruction may be used by some surgeons in certain situations due to hostile urethral tissues, in the majority of cases, LS is amenable to a single-stage reconstruction with highly favorable results. Additionally, reconstruction of long-segment urethral strictures is not only about restoring voiding function but also preserving sexual function in all its aspects, such as erection, ejaculation, and orgasm as well as guaranteeing good penile cosmesis.

Current surgical options employed are associated with reasonable success rates and may include a single- or a multiple-stage reconstruction, with the use of a flap, a graft, or a combination of both, and lastly, in extreme circumstances a perineal urethrostomy may offer the best solution for the patient who does not wish to go the extra (long) mile.

## 2. Materials and Methods

A review of the international literature was conducted using MEDLINE/PubMed database and Google Search, using keywords as “complex urethral stricture,” “long segment urethral stricture,” “panurethral,” “lichen sclerosus,” “oral mucosa,” and “urethroplasty.” We included in the review only articles published in the English language from 1990 to 2015.

## 3. Epidemiology, Etiology, and Pathogenesis

Generally speaking, male urethral stricture is a common disease worldwide and has been so for centuries. The first known description of urethral dilatation is credited to Shusruta more than 600 years BC [11]. In the 19th century, expert opinion estimated an incidence of 15–20% in the adult male population [12]. In the 21st century in the UK NHS more than 16,000 men required hospital admission annually due to urethral stricture and more than 12,000 of these admissions ended up necessitating surgical treatment with more than £10,000 million [12]. The estimated prevalence in the UK averages 10/100,000 young males doubling this figure by the age of 55 years and rising to over 100/100,000 in males over 65 years. In the USA male urethral stricture accounted for about 5,000 inpatient visits and 1.5 million office visits annually between 1992 and 2000. The incidence was estimated to be approximately 0.6% in susceptible populations [13]. The estimated costs to the medical system for male urethral disease in the USA surpassed US$ 190 million in 2000 [13]. However, there are no direct measures to assess the true incidence of urethral stricture disease worldwide, much less so for panurethral stricture disease in particular. A recent study, including 268 patients, reported panurethral or multifocal anterior urethral stricture in a total of 36 patients (13.4%). However, in a more recent retrospective analysis of all strictures that had been treated surgically at a single institution, the vast majority of strictures were anterior (92.2%) with panurethral strictures totalling 4.9% [14].

Urethral stricture disease can have a profound impact on quality of life, including sexual life, as a result of a number of complications associated with urinary obstruction, such as infection, bladder calculi, urethral diverticulum, fistulation, sepsis, and ultimately chronic renal failure.

The etiology of long-segment or panurethral strictures may vary in industrialized and developing countries. Today, in industrialized countries, most urethral strictures in general have iatrogenic or idiopathic origin [2, 3]. Iatrogenic causes include urethral catheterization, cystourethroscopy, transurethral resection, and previous urethral surgeries. Other causes include idiopathic, trauma, infection/inflammation, and lichen sclerosus. In the developing world, the most common cause of panurethral stricture is genital lichen sclerosus (LS) [6]. Although less frequent, gonorrhea still remains an important cause of long-segment strictures in the developing world.

The pathogenesis of long-segment or panurethral stricture disease has not been widely studied. Historically, and although it is an important cause in some regions of the developing world, infection was blamed as the main cause of urethral stricture [15]. However, it must follow a similar pathogenic process as other types of urethral stricture, that is, injury to the epithelium of the urethra and underlying corpus spongiosum, ultimately leading to fibrosis during the healing process. Excepting a traumatic cause when the urethral lumen is obliterated, corpus spongiosum deep to the urethral epithelium is replaced by dense fibrous tissue and the normal urethral pseudostratified columnar epithelium being replaced by squamous metaplasia [16–18]. Metaplastic change can also occur proximal to a stricture, due to chronic distension under pressure of voiding [12]. Small tears occurring repeatedly in the metaplastic tissue result in focal urinary extravasation, which in turn leads to a fibrotic reaction within the spongiosum. Initially, this fibrosis can be asymptomatic, but, over time, the scar or fibrotic plaque produced can enhance the narrowing of the urethral lumen, resulting in symptomatic urinary obstruction.

The pathology of a urethral stricture is characterized by changes in the extracellular matrix of the spongiosal tissue and replacement of the normal connective tissue by dense fibrosis associated with a decrease in the ratio of type III to type I collagen and a significant decrease in the smooth muscle and nitric oxide content in the strictured urethral tissue [19, 20].

**Figure 1:**
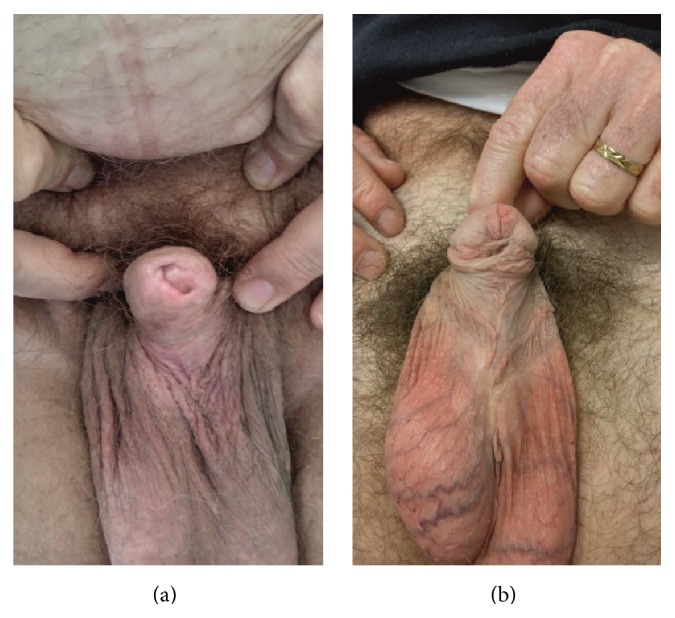
Lichen sclerosus of the glans and prepuce (a) and hypospadias cripple (b). Both patients with panurethral stricture.

The pathology of lichen sclerosus in inducing urethral stricture is different. LS is a chronic, progressive, inflammatory process which in the male can involve foreskin, glans, and anterior urethra. The etiology is for the most part unclear, although it has been associated with an autoimmune reaction and a genetic pattern. However, an infectious cause has been suggested [21]. This is an atrophic rather than a proliferative process that usually originates in the foreskin or glans as diffuse or patchy plaques of white discoloration giving the glans a characteristically mottled appearance (Figure 1). It can progress further to include the meatus, fossa navicularis, penile urethra, and eventually the bulbar urethra, resulting in a long-segment or panurethral stricture disease [7, 8]. It remains unclear whether LS-induced urethral strictures develop as a consequence of extension of glandular disease into the penile urethra or whether they result from chronically obstructed voiding or instrumentation, or both [22]. Long-segment urethral strictures, as any anterior urethral stricture, typically occur following trauma or infection, but mostly from iatrogenic causes, especially urethral catheterization, dilatation, and endoscopic manipulation, or may be idiopathic. Nonetheless, LS has been reported as the most frequent cause of this type of stricture, especially in India [6, 8, 9].

## 4. Diagnostic Evaluation

A critical initial pitfall in the diagnostic evaluation is not to fully understand and properly diagnose the stricture as being panurethral. Symptomatic stricture disease typically presents with progressive obstructive voiding complaints, such as a weak stream, frequency, incomplete emptying, terminal dribbling and straining, or complications of an obstructive voiding syndrome, such as recurrent tract infections, epididymitis, haematuria, and bladder stones. Symptomatic evaluation should be best formalized using a validated questionnaire, such as the AUA symptom index [24, 25].

Physical examination may be vague and uneventful in some cases. Nonetheless, the penis should always be carefully examined for scars related to previous surgery, penile malformations, signs of LS, or associated penile cancer. Careful attention should also be drawn to palpation of the spongiosum and genital area in general. The mouth should also be carefully inspected, particularly if an oral mucosa graft is planned.

**Figure 2:**
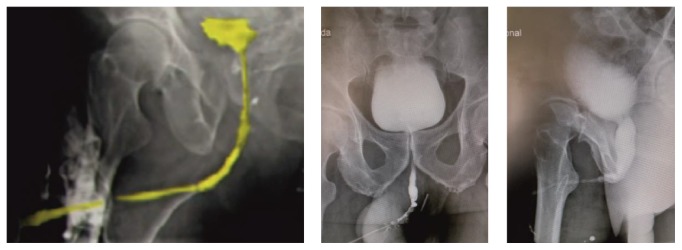
Retrograde and voiding urethrogram of panurethral stricture disease.

Uroflowmetry, ultrasonography, and cystourethroscopy may be important adjuncts in the diagnosis of panurethral stricture disease, but the most critical is retrograde urethrography (RUG) and voiding cystourethrography (VCUG). The latter tests determine the location, length, and severity of the stricture in great detail (Figure 2). Endoscopy can give an idea of the elasticity and appearance of the urethra, especially following previous urethroplasty(ies). Ultrasonography can be used to determine the length and degree of fibrosis and eventually influence the operative approach [26]. Although ultrasonography seems to provide important additional information during preoperative evaluation, it has not gained the expected widespread popularity. This may be due to its relatively limited usefulness in the more proximal bulbar urethra, where the distance between the ultrasound probe and the target area surpasses its resolution accuracy.

It is of paramount importance that these imaging modalities ensure that all diseased portions of the urethra are included in the repair. Often, the narrowing of the lumen can be fairly uniform, with spots of more severe reduction in caliber. Thus, a panurethral stricture can be erroneously interpreted as just a short stricture and the other less severe areas underestimated as being of “normal” caliber. To avoid this diagnostic error, some authors have suggested that if the urethral lumen does not expand to ≥ 8 mm in diameter on imaging, then it is probably stenosed. Sometimes, it may be necessary to proceed to a full examination under anesthesia with endoscopy and bougienage and retrograde urethral imaging [27].

## 5. Surgical Reconstruction

In rare instances, where symptoms are not particularly troublesome, surgical treatment may not be necessary. In the majority of patients, both urethral dilatation and direct vision internal urethrotomy are inappropriate and, therefore, have no place in the treatment of panurethral stricture disease. At the other extreme end of the spectrum of this disease, typically patients who have undergone multiple failed surgical attempts, particularly when associated with significant comorbidity, might prefer a definitive perineal urethrostomy or even opt a simple suprapubic cystostomy catheter.

Panurethral stricture disease is definitely a complex subset of urethral stricture disease. Defining “panurethral” has been a matter of debate. This has implications in the interpretation of the literature as there is no homogeneity in the study populations. In a recent multi-institution study including 466 patients, long-segment or panurethral stricture was defined as any single stricture or multifocal diseased areas of the penile and bulbar (anterior) urethra measuring ≥ 8 cm in length [23]. Several surgical reconstructive procedures have been described to address this full-length anterior urethral strictures (Table 1). When planning the surgical treatment of panurethral stricture disease, some surgeons have concerns of whether to select a one- or two-stage operation and, if a one-stage operation is chosen, whether adequate transfer tissue for reconstruction is available. Panurethral stricture disease associated with LS has been successfully treated with a single-stage repair and OM onlay grafting. Indeed, the authors' experience has clearly shown that it should be preferred over a multistage approach, which in their opinion has no role in the surgical treatment of genitourethral LS [6, 23]. The main arguments are the high failure rate; the fact that genitourethral LS is a penile skin disease and, lastly, that staged operations will allow ingrowth of the disease into the urethra. In less common instances, where there is significant urethral narrowing with an unsalvageable plate, after multiple failed previous repairs, or if the stricture disease is associated with infection, abscess or calculi, a two-stage marsupializing procedure, like the Johanson procedure, may be preferable. In the majority of cases, substitution urethroplasty is the rule. Substitution urethroplasty can be performed using a flap, a graft, or sometimes a combination of both.

### 5.1. Flaps

Several flaps have been described and used in panurethral stricture reconstruction. In 1993, McAninch described the* circular fasciocutaneous penile flap* for the reconstruction of extensive urethral stricture [28]. Circular fasciocutaneous penile flap originates on the distal penis and uses Buck's fascia as the major vascular supply. He reported his results with the use of this flap for 1-stage reconstruction of complex anterior urethral strictures involving long penile and also bulbar urethral strictures in 66 men [29]. The stricture length measured up to 24 cm (average 9.08 cm). The flap was used as an onlay procedure and tubularized flap for urethral substitution. In some cases, additional adjunctive tissue transfer and proximal graft placement were required. Initial success rate was 79%, rising up to 95% after an additional procedure. Recurrent strictures occurred usually at the proximal and distal anastomotic sites. The penile circular fasciocutaneous flap reliably provided 12–15 cm of length for reconstruction in most patients, although approximately 90% had been previously circumcised. The less favorable results were seen in patients after flap tubularization for urethral replacement. The McAninch technique is worldwide considered as a reliable surgical option for panurethral strictures and numerous publications are available in the literature about its use. A major advantage of the McAninch flap is its versatility, as it can be utilized in all areas of the urethra, from the membranous area to the external meatus [29, 30]. Because of compartment syndrome noted in 2 different cases due to prolonged exaggerated lithotomy position that usually occurs if the patient remains in this position more than 5 hours, the authors begin the operation with flap harvesting with the patient in the supine position, thereby reducing exposure to the lithotomy position by 2-3 hours.

The* Q-flap* is a modification of the McAninch circular penile fasciocutaneous skin flap. It is so called because it incorporates an additional midline ventral longitudinal penile extension, thus resembling the letter Q. Similar “hockey-stick” flap configurations have also been described by Quartey [31]. Morey et al. reported their experience with the Q-flap in 15 patients with a mean stricture length of 15.5 cm (range 12–21) who underwent single-stage urethral reconstruction. All patients had a prepuce and the flap was harvested with the patient initially supine to avoid compartment syndrome [32]. The flap is outlined with the penis on stretch and the penis degloved, meticulously preserving the blood supply on the tunica dartos pedicle. The Q-flap is sewn into place after ventral urethrotomy as an onlay flap with running 4-0 absorbable suture, similar to the McAninch flap procedure. The fossa navicularis is typically reconstructed through a glans-wings or a glans-preserving technique. Once the pendulous portion of the onlay flap is sewn in, the patient is repositioned into the lithotomy position and the flap is transferred to the perineum through a scrotal tunnel wide enough to accommodate loose passage of the flap. The potential major advantage of these flap procedures is to allow a single-stage reconstruction of long-segment and complex strictures and to avoid the need for additional, morbid, time-consuming tissue transfer techniques.

These two procedures are extremely labor-intensive and are among the most difficult and tedious in reconstructive urology. A common complication with the above two flaps, particularly with unexperienced surgeons, is necrosis of penile skin proximal to the flap [29, 30]. In some instances, this penile skin necrosis may lead to wound infection and ultimately to disruption of the flap and necrosis.

In 1997, Gil-Vernet et al. described another type of flap for urethroplasty, the* biaxial epilated scrotal flap* [33]. They used this flap, which measured up to 20 × 2.5 cm, to reconstruct the entire anterior urethra from the bulbomembranous urethra to the external meatus. This flap consists of scrotal skin, dartos, external spermatic fascia, cremasteric fibers and fascia, internal spermatic fascia, and scrotal septum. Tunica vaginalis is not included. Vascular anastomoses between cremasteric (deep) and scrotal (superficial) blood supply plexuses are included in the flap and hence biaxial flap. The authors used this technique in 37 men including 10 with panurethral stricture disease. Two of these 10 patients failed due to graft shrinkage, necessitating perineal urethrostomy. There were also problems with incorrect scrotal skin epilation leading to sclerosis, vascular lesions, and penile ventral curvature. Nonetheless, the authors considered this flap technique ideal for urethral reconstruction from the penoscrotal angle to the prostatic apex. Because of anatomical proximity, good tissue availability, and potentially good tolerance to contact with urine due to abundance of sebaceous glands, this is always the authors' first option for bulbomembranous urethroplasty. They also believe that scrotal skin flap is less likely to develop lichen sclerosus as compared to penile skin. Despite all the potential advantages mentioned by the authors, epilation, deepithelialization, and flap mobilization may not be so straightforward. Epilation is an extremely time-consuming process. Although flaps with their own blood supply would be more appropriate in severely fibrotic urethral beds, such as after previously failed urethroplasties, several problems with postvoid dribbling of urine, ejaculatory dysfunction, and flap outpouching or pseudodiverticulum formation are truly troublesome and impact on quality of life [30]. It should be kept in mind that, in general, the use of skin flaps for urethral reconstruction is more technically demanding than substitution urethroplasty. In a study by McAninch and Morey, for patients with an average stricture length of 9 cm, the initial overall success rate of the fasciocutaneous flap reconstruction was 79%. Recurrent stricture rate was noted in 13% of onlay grafts and in 58% of tubularized repairs [29].

### 5.2. Grafts

The use of grafts in urethral reconstruction has become a more popularized surgical option worldwide. Theoretically, grafts in general are inherently less reliable because they need to be vascularized. However, they are quick and relatively easier to harvest and deploy. There are several studies of both flaps and grafts showing similar restricture rates [34]. Therefore, in the authors' opinion, a graft should be the procedure of choice due to its simplicity and speed by which it can be harvested and deployed, since the restricture rate is similar. There may be specific indications favoring a flap rather than a graft: revision surgery following multiple failed attempts, any cause of local devascularization such as irradiation or severe peripheral vascular insufficiency, and local infection, all of which hamper the ability of a graft to take. In summary, a graft repair is preferred due to the reasons mentioned above. Both grafts and flaps contract, although full-thickness flaps tend to contract less than split-thickness flaps and grafts, and patch grafts do better than tubed grafts, which may imply a two-stage procedure if a circumferential reconstruction of the urethra is necessary.

**Figure 3:**
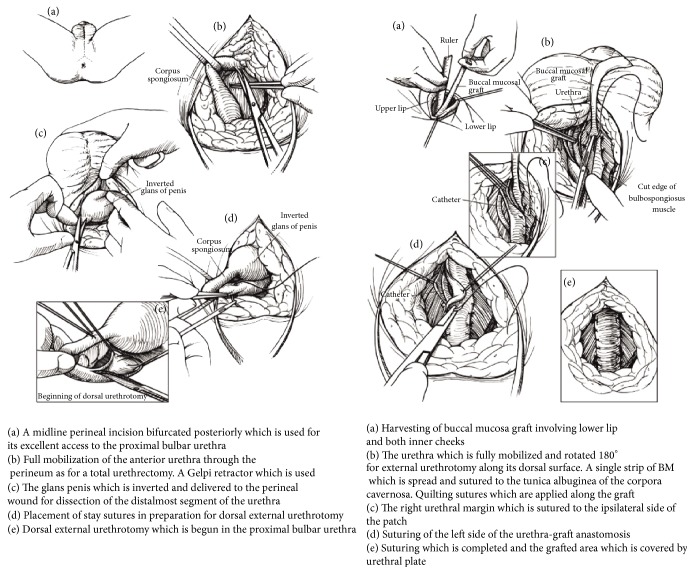
Schematic representation of the Kulkarni operation.

**Figure 4:**
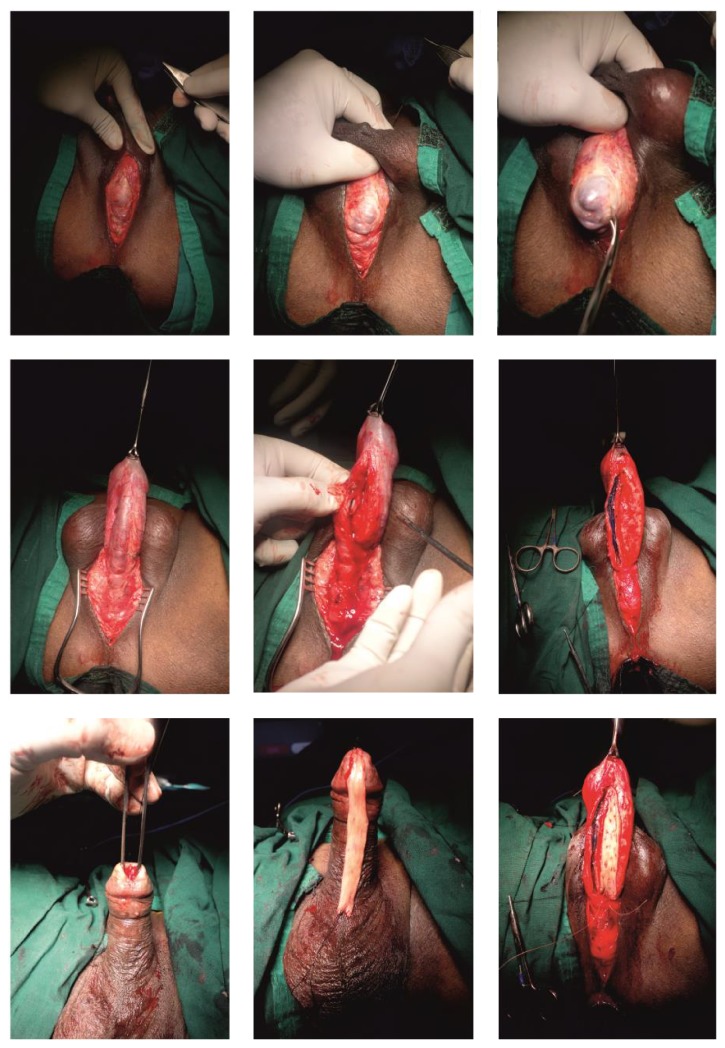
Kulkarni operation.

The widespread popularity of* oral mucosa* in urethral reconstruction has similarly allowed the introduction of new techniques in long-segment and panurethral stricture repair. In 2000, Kulkarni et al. first described the use of long oral mucosa grafts to repair the entire anterior urethra through a simple perineal incision in a single stage, thus preserving the penile components, their anatomy, function, and cosmesis [35] (Figure 3). In 2009, the same authors described a modification of their original technique, suggesting a minimally invasive procedure with dissection of the urethra from the corpora cavernosa along one side only, thus preserving the entire neurovascular supply to the urethra [36] (Figure 4). Buccal mucosa graft urethroplasty has been used for long anterior urethral strictures by several authors following the initial report by Kulkarni et al. in 2000 [37–40]. All these authors have reported favorable results at short- and medium-term follow-up with acceptable complication rates. In 2004, Gupta et al. described a technique of dorsal graft placement by ventral sagittal urethrotomy and minimal-access perineal approach and used this technique in patients with anterior urethral stricture, including 2 with panurethral stricture disease [40]. In the Kulkarni technique, the whole anterior urethra is repaired by a single perineal incision, single technique, and single substitute material (Figure 4). In a retrospective study including 117 patients with panurethral stricture disease treated from June 1998 to December 2010, the overall success rate was 83.7%. Mean stricture length was 14 cm and median follow-up was 59 months. Most recurrent strictures occurred at the proximal anastomotic site and none of these was a full-length recurrence [6]. The major advantage of this technique is that it is minimally invasive and performed in one stage. It also avoids the psychological trauma of 2 (or more) operations and the need of living for 6 months with bifid scrotum after staged procedures. Additionally, because it is a one-side dissection the risk of injury to the neurovascular bundles to the penis and urethra is minimal. This procedure is carried out through the perineum, avoiding a penile scar, and does not lead to a hypospadiac meatus.

More recently, some authors have described the use of* lingual mucosa* in urethroplasty [41–45]. The graft characteristics of lingual mucosa are similar to those of buccal mucosa (cheek and lip) [41, 42]. Lingual mucosal graft was used as the sole graft in 18 men with long anterior urethral strictures by Das et al. [41]. Most cases were etiologically associated with LS or infection. Overall success rate was 83.3%. However, separate results regarding panurethral strictures were not given. A particular advantage of lingual mucosa is that it can be harvested in continuity across the midline with the opposite side of the tongue, allowing a graft of sufficient length for panurethral strictures.

Prepuce and penile skin in the form of flaps or grafts are recognized alternatives for this type of reconstruction and are mentioned in the Table 1. In experienced hands, oral mucosal grafts measuring 10 × 1.5 cm can routinely be harvested from each inner cheek. If necessary, lingual grafts can be harvested in addition. A great number of our patients who have LS have scarred prepuce and glans and already had circumcision. In LS, no form of genital skin can or should be used. Preputial/distal penile skin graft was described for dorsal onlay anterior urethroplasty. In most studies, panurethral stricture patients were a minority [46, 47]. Most failures occurred if the skin graft was placed onto the penile urethra. Although previous circumcision did not preclude the use of penile skin, buccal mucosa was recommended as the best choice if the shaft skin was not abundant [46]. Postauricular skin has also been used as a good alternative for men with panurethral strictures with high success rate [48–50]. Postauricular skin is thin and has a dense subdermal plexus, and, therefore, graft take and functional outcomes are superior to other nongenital skin grafts. However, Andrich and Mundy cautioned that no skin graft should be used for urethroplasty in LS patients. LS is a skin disease and can also affect any skin graft in due course [49].

Another subject of controversy is the location for graft placement. Ventral graft placement, particularly in the pendulous urethra, is usually associated with poorer results. In the bulbar urethra, similar results can be expected, as long as ventral grafting is not used for long and complex strictures. A flap or a two-stage procedure is advocated by some authors for these strictures [46]. Dorsal graft placement usually produces the best outcomes and, therefore, is the method of choice in panurethral strictures [6, 23, 51–53]. Although doubled-sided dorsal plus ventral oral mucosa grafting has also been suggested for bulbar urethroplasty, the authors did not recommend its use for strictures measuring more than 4 cm in length [54]. Therefore, this technique is not indicated in long-segment or panurethral stricture disease.

Colonic mucosa has been employed for the reconstruction of panurethral stricture disease [55]. This graft is harvested from sigmoid colon using a laparoscopic approach or by a lower abdominal paramedian incision. Full-thickness grafts of 12 to 15 cm in length of sigmoid colon mucosa can be obtained and the colon continuity is immediately restored by an end-to-end anastomosis. An unstretched colonic mucosa graft is trimmed and sized to an appropriate individual need (ranging from 15 to 22 cm in length and 3 cm in width) and is tubularized over a 16 to 18 Fr fenestrated or fluted silicone catheter with interrupted 5-0 absorbable suture to create a neourethra. An end-to-end anastomosis is performed between the neourethra and the proximal end of the native urethra. The distal end of the neourethra is pulled through the glans tunnel to form the neomeatus. Xu et al. reported their experience with 35 patients who underwent colonic mucosal graft urethroplasty for complex, long-segment urethral strictures, ranging from 11 to 21 cm in length (mean 15.1). Five (14.2%) of these patients developed recurrent strictures. However, 3 of the recurrences were not related to the urethroplasty. Therefore, they concluded that tubularized urethroplasty using colonic mucosa grafts was successful and had a lower recurrence rate than patch urethroplasty. Nonetheless, this procedure needs further investigation and confirmation and, therefore, should be reserved as an alternative in complex patients where other options are not available or possible.

### 5.3. Combination of Flaps and Grafts

The exclusive use of long flaps for complex or panurethral strictures may be a technically challenging ordeal and are usually associated with long operating times and morbidity due to positioning and the surgical procedure itself. Furthermore, sufficient length of skin flaps may not be available, particularly in circumcised men or if LS is present. In such cases, a reasonable treatment option is to combine a shorter flap with a graft, and the graft placed proximally in the bulbar urethra [51]. A penile circular fasciocutaneous flap combined with an oral mucosa graft placed proximally was used by Wessells et al. in 7 patients with a mean stricture length of 18.3 cm [56]. The mean flap length was 12.6 cm (range 10–15) and mean graft length was 6.2 cm (range 3–9). The overall success rate was 88% at 16 months follow-up. Unfortunately, the authors did not mention results specific to panurethral strictures separately. The authors emphasized the importance of avoiding tubed reconstructions as these are associated with high risk of restricture and other flap-related complications.

Oral mucosa has become the graft material of choice for substitution urethroplasty, but at times it may be insufficient to completely reconstruct a long-segment or panurethral stricture. The combined use of oral mucosa and a genital skin flap has proved to be a reliable and durable alternative for single-stage reconstruction of long-segment or panurethral stricture disease [51].

### 5.4. Staged Procedures

At present, the majority of uncomplicated anterior urethral strictures can be successfully managed with a single-stage procedure. However, complex strictures associated with adverse local conditions, such as extensive scarred tissue formation of the urethra, infection, fistulation, prior multiple failed urethral reconstruction attempts, totally obliterated residual urethra, graft or flap-related factors, or following heavy irradiation, represent a challenge and are more appropriately treated with a staged procedure. A staged reconstruction may also be indicated in some long urethral strictures. All these situations are associated with unhealthy, poorly vascularized, and inelastic urethral and neighbouring tissues for urethral reconstruction. Although LS can be managed with a single-stage reconstruction, in some cases a staged procedure may be a reasonable option, as it may have a beneficial impact on the natural history of the disease [57–60]. A perineal urethrostomy for urinary diversion avoids continuous extravasation into the corpus spongiosum and promotes quicker and better urethral tissue healing.

**Figure 5:**
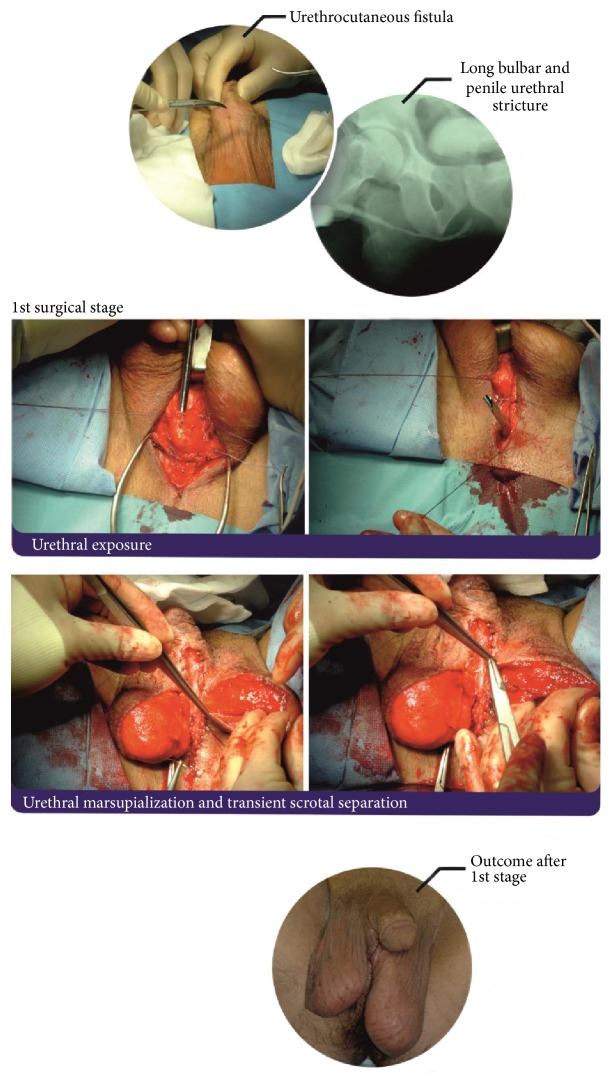
First stage of Johanson reconstruction with OMG inlay of panurethral stricture.

**Figure 6:**
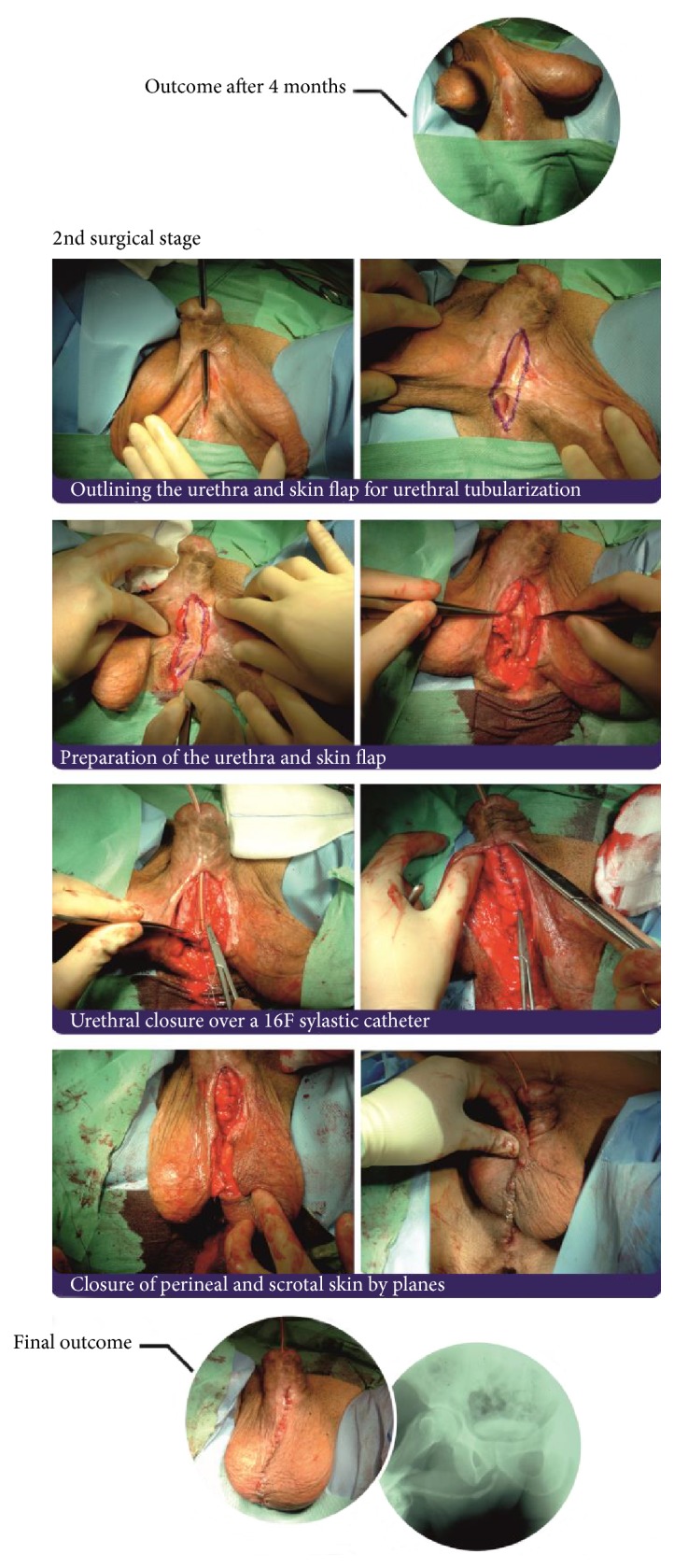
Second stage and closure.

The classical two-stage method was developed in the 1950s by Johanson [61]. The Johanson procedure is based on marsupialization of the strictured urethra, followed by a second surgical stage approximately 4–6 months after the first stage has healed (Figures 5 and 6). In the past, scrotal or perineal skin was used for urethral reconstruction. The great achievement of Johanson's technique was its use in all types of strictures, apart from initiating an era of urethral reconstructive surgery. The drawbacks of this technique resulted from the use of hair-growing scrotal and perineal skin, which lead to chronic urinary tract infection, abscesses, lithogenesis, fistulation, sacculation, and diverticula formation in the reconstructed urethra.

In the 1980s, Schreiter reported a two-stage mesh graft procedure in an attempt to avoid the use of scrotal or perineal skin by using a hairless skin graft which is transferred to a two-stage procedure [62, 63]. Although this technique can be employed in every type of stricture, apparently its best indication is in complex strictures, especially associated with severe tissue scarring and absence of healthy penile skin for urethral reconstruction.

More recently, other authors have reported on a two-stage Johanson-type urethroplasty with oral mucosa grafting for anterior urethral strictures. For penile urethral strictures, Patterson and Chapple favor a two-stage procedure with dorsal onlay oral mucosa grafting after complete excision of the scarred urethra [64].

Staged reconstructions are associated with significant inconvenience to some patients, exposing them to an increased risk of morbidity due to multiple general anesthetics. Additionally, revision is common after two-stage operations and in one series half of the patients ended up needing a three-stage repair [65].

### 5.5. Tunica Albuginea (Monseur) Urethroplasty

In 1969, Monseur described a procedure by which a neourethra was created and its lumen continuity was maintained by the tunica albuginea through a supraurethral or subcavernosal groove without the need of a graft or flap [66]. Recently, there has been some renewed interest in this technique and various reports have been published on the use of Monseur's tunica albuginea urethroplasty for short- and long-segment urethral strictures with acceptable success rates [67, 68]. The authors reported on the utility of this technique in cases where oral mucosa urethroplasty is not feasible due to lack of healthy oral mucosa associated with tobacco chewing or need of very long grafts to bridge panurethral strictures [69]. The authors described some similarity with the tubularized incised plate (TIP) urethroplasty described by Snodgrass and Bush, where the tunica exposed after incision of the urethral dorsal plate forms the roof of the neourethra and has stood the test of time for that purpose [70]. The authors argue that flap procedures are considered extremely labor-intensive, tedious, and among the most difficult in reconstructive urology [32]. Oral mucosa graft procedures, although very successful in medium-sized strictures, may not be feasible in very long strictures. Because some studies have shown that, even in dorsal onlay grafting, oral mucosa and penile skin grafts have shown similar results, while both proving superior to flaps, these authors concluded that it is not the type of graft, but rather the site of graft that is ultimately responsible for the success of the procedure [71]. Other studies have reported that a ventral onlay graft has a significant disadvantage over a dorsal onlay. It is claimed that complications are decreased if the graft is placed dorsally over the urethral groove [72]. Based on the concept advocated by Monseur, Barbagli et al. introduced the dorsally placed (onlay) graft technique and postulated that dorsal graft placement is superior as it allows better mechanical support for the graft and a richer and predictable vascular blood supply for the graft from the underlying corpora cavernosa [73, 74]. So if it is assumed that dorsal onlay grafts yield results better than ventral, then it must be the site of graft placement rather than the type of graft material that is ultimately responsible for the better success rates [75]. Lastly, the authors claim that Monseur's tunica albuginea urethroplasty is easy to perform, with short learning curve, without graft morbidity, requiring less time and resources, and success rates are comparable to oral mucosa urethroplasty. Tunica albuginea appears to be sufficient to allow regrowth of urethral epithelium and a patent distensible lumen if proved by urethrosccopic biopsy. However, we think that further studies are necessary.

## 6. Success and Complications

Generally, urethroplasty has excellent success rates and far exceed those found with direct vision internal urethrotomy. Serious complications following urethroplasty are relatively uncommon, 3% of them occurring in the early postoperative period and 18% in a late follow-up period [76]. Most reports in the literature contain heterogeneous data, that is, different types of strictures treated by different modalities and surgeons. When complications are mentioned, they are mixed for all the procedures. Another pitfall found in the literature is in comparing success rates in different series, as these have variable definitions of treatment failure. Therefore, consensus in reconstructive urology needs to be established in the future.

Generally, complications of urethroplasty are directly related to location of stricture, length of stricture, operative technique, and type of transfer tissue employed (Table 2). Complications after urethroplasty can be divided into major and minor groups, occurring early or late. Most minor complications are usually mild and temporary and may be amenable to simple corrective procedures. Major complications are usually severe and complicated and result in failure of urethroplasty. In this review we will focus on complications associated with the reconstructive procedures of long-segment anterior urethral or panurethral strictures (Table 3).

Oral mucosal grafts are now considered the standard substitution material for urethral surgery. Surgical procedures involving oral mucosa onlay have better success rates and less morbidity compared with fasciocutaneous flaps [23, 46, 73, 74, 76]. One report has mentioned complication rates of fasciocutaneous flaps between 3% and 56% [77]. In a multi-institutional study, Warner et al. reported on the complication rates of different surgical techniques to repair long-segment and panurethral strictures [23]. The complication rate was higher in the fasciocutaneous cohort compared with those without a flap (32% versus 14%, resp.; *P* = 0.02). In this review, a 2-stage Johanson urethroplasty was not as successful as the buccal mucosal graft procedure (BMG) (64% versus 82.5%, resp.). It was found that 2-stage Johanson urethroplasties performed with skin had a higher failure rate than those performed with a BMG (66.7% recurrence rate versus 28.3%, resp.). Meticulous follow-up of patients after long-segment or “panurethroplasty” may show an important percentage of early and late complications. Perineal neuralgia or neuropraxia is a well-known complication of bulbar and posterior urethroplasty, or any surgery performed in the exaggerated lithotomy position (i.e., radical perineal prostatectomy and urorectal fistula repair) [78–80]. The most common position-related complications of complex urethroplasty include superficial peroneal nerve neuropraxia, rhabdomyolysis, and lower extremity compartment syndrome. Although several causes of neuropraxia have been identified, the mechanical nerve compression seems to be the most common. It usually resolves spontaneously within 6–8 weeks. Recent studies have reported much lower position-related complication rates not exceeding 3% due to shortening of overall lithotomy position and meticulous protocol of patient protection during this type of surgery. In the authors' personal series, the severe neuropraxia rate has been in accordance with these reports.

Although most complications are minor, with little impact, and easily corrected, they seem to occur in a higher number than previously published (40%) [76]. These complications are important to the patient and should be discussed in the counseling before surgery.

## 7. Impact on Sexual Function

Impairment of male sexual function (penile sensation, erectile, and ejaculatory dysfunctions) are usually underreported. However, this scenario has been changed recently. In 2001, Coursey et al. reported a study on erectile function after anterior urethroplasty based on a questionnaire evaluation of erectile dysfunction. ED occurred in 19% of patients after OMG and 27% after anastomotic urethroplasty. Although he postulated that men with a long stricture might be at increased risk for transient erectile changes, the overall postoperative sexual dysfunction rate was no higher than circumcision [81]. Based on validated inventory questionnaires, such as the International Index of Erectile Function (IIEF-5) or the O'Leary Brief Male Sexual Function Inventory (BMSFI), the majority of the studies published recently have not shown that urethral reconstructive surgery impairs erectile function and sexual drive. Ejaculatory function was even improved in the younger ages [82–86]. Although erectile dysfunction has been associated with urethroplasty operations, its incidence is largely unknown. A 1% incidence of* de novo* erectile dysfunction after anterior urethroplasty was found in a meta-analysis study by Blaschko et al. However, in most cases the erectile dysfunction was transient and resolved within the first 12 months [85]. Another study reported an incidence of transient erectile dysfunction after anterior urethroplasty in approximately 40%, although recovery was observed in most by 6 months [83]. In 2006, the same authors had described a relationship between older age and a higher incidence of erectile dysfunction after surgery. Nonetheless, overall, men had not reported a decline in erectile dysfunction or sexual drive after urethroplasty [82]. In 2015, Xu et al. published a study dealing specifically with the impact of erectile dysfunction on complex panurethral stricture disease. They concluded that the surgical reconstruction with the use of grafts (buccal, lingual, and colonic mucosa) had limited effect on erectile function. The only adverse factor was extension of the stricture to posterior urethra, in which case an impairment was observed [86]. Ejaculatory dysfunction was reported in patients after ventrally placed flaps or grafts, possibly due to urethrocele formation [87]. However, no ejaculatory dysfunction has been reported in patients after dorsal onlays [77, 87].

## 8. Conclusion

One-stage repairs with BMG offer an excellent option for patients with long-segment and panurethral stricture disease. In cases with obliterative or absent urethral plate, a 2-stage Johanson urethroplasty with BMG offers a viable alternative. In cases of LS, 1-stage BMG has better outcomes than a 2-stage repair. If BMGs are not available, FC flaps offer similar success; however, these are associated with higher rates of complications. Skin grafts should be avoided, unless no alternatives exist. Finally, the valuable role of PU cannot be understated in the setting of multiple failed urethroplasties.

The options currently available to reconstruct the urethra are in permanent development and attention should be focused on both old and new concepts. No surgical technique should compromise penile length, cause chordee, and affect cosmesis. Oral morbidity should be given attention after OMG to avoid permanent late sequelae in mouth function. Critical attention should also be given to sexual function as any urethral reconstructive method can eventually cause its occurrence.

## Figures and Tables

**Table 1:** Options for surgical reconstruction of long-segment and panurethral strictures.

	Flaps
	Circular fasciocutaneous penile flap (McAninch flap)
	Q-flap and variants (Quartey and Jordan)
	Biaxial epilated scrotal flap (Gil-Vernet)
	Grafts
	Oral mucosa (cheek, tongue, and lower lip)—Kulkarni technique
	Postauricular skin (Wolf)
	Penile and preputial skin
	Bladder mucosa
	Colonic mucosa
	Combination of flaps and grafts
	Staged procedures
	Johanson technique and variants
	Schreiter's mesh graft technique
	Tunica albuginea (Monseur) urethroplasty
	Perineal urethrostomy

**Table 2:** Major and minor complications of “panurethroplasty”^*∗*^.

Major	Minor
Early	Early
Hematuria	Oral numbness
RUG leak	Drooling when eating or Speaking
Oral discomfort	Speech impairment
Wound dehiscence	Perineal hypoesthesia
Wound tightness	Scrotal hyperesthesia
Epididymitis	Stensen's duct squirting
Penile ecchymosis	Penile pain
Penile swelling	Penile shortening
Penile skin ischemia/necrosis	Postvoid dribbling
UTI	Stress incontinence
Wound infection	Urine splaying
Late	Late
Rectal injury	Recurrent stricture
Urosepsis	Sexual dysfunction
	Chordee
	Fistulation

^*∗*^Generally, similar and common to any urethroplasty.

**Table 3:** Complications by most common techniques for pan-urethroplasty.

Type of surgery	Early	Late	Recurrence
FC flap	Transient pain and numbness Fistula (resolved)	Fistula	37.5%
OMG	UTI Penile edema Bleeding	Chordee Fistula ED Oral and lip discomfort numbness Cold glans	17.5%
Second-stage Johanson	Wound dehiscence UTI Scrotal abscess Penile numbness Epididymitis	ED Graft contracture Fistula Chordee Cold glans	35.7%
PU and definitive 1st-stage Johanson	Wound dehiscence UTI Transient pain and numbness	Chordee Fistula	24.1%
FC flap + graft	Wound hematoma PE Penile skin ischemia	Fistula Chordee	23.5%

FC: fasciocutaneous; OMG: oral mucosal graft; UTI: urinary tract infection; ED: erectile dysfunction; PU: perineal urethrostomy; PE: pulmonary embolism. Adapted from [23].
